# Increased SNAIL expression and low syndecan levels are associated with high Gleason grade in prostate cancer

**DOI:** 10.3892/ijo.2014.2254

**Published:** 2014-01-10

**Authors:** CRISTIAN E. POBLETE, JUAN FULLA, MARCELA GALLARDO, VALENTINA MUÑOZ, ENRIQUE A. CASTELLÓN, IVAN GALLEGOS, HECTOR R. CONTRERAS

**Affiliations:** 1 Physiology and Biophysics Program, Institute of Biomedical Sciences, Faculty of Medicine, University of Chile, Santiago, Chile; 2 Pathological Anatomy Service, Clinic Hospital, University of Chile, Santiago, Chile

**Keywords:** prostate cancer, SNAIL, syndecans, epithelial-mesenchymal transition

## Abstract

Prostate cancer (PC) is a leading male oncologic malignancy wideworld. During malignant transformation, normal epithelial cells undergo genetic and morphological changes known as epithelial-mesenchymal transition (EMT). Several regulatory genes and specific marker proteins are involved in PC EMT. Recently, syndecans have been associated with malignancy grade and Gleason score in PC. Considering that SNAIL is mainly a gene repressor increased in PC and that syndecan promoters have putative binding sites for this repressor, we propose that SNAIL might regulate syndecan expression during PC EMT. The aim of this study was to analyze immunochemically the expression of SNAIL, syndecans 1 and 2 and other EMT markers in a tissue microarray (TMA) of PC samples and PC cell lines. The TMAs included PC samples of different Gleason grade and benign prostatic hyperplasia (BPH) samples, as non-malignant controls. PC3 and LNCaP cell lines were used as models of PC representing different tumorigenic capacities. Semi-quantitative immunohistochemistry was performed on TMAs and fluorescence immunocytochemistry and western blot analysis were conducted on cell cultures. Results show that SNAIL exhibits increased expression in high Gleason specimens compared to low histological grade and BPH samples. Accordingly, PC3 cells show higher SNAIL expression levels compared to LNCaP cells. Conversely, syndecan 1, similarly to E-cadherin (a known marker of EMT), shows a decreased expression in high Gleason grades samples and PC3 cells. Interestingly, syndecan 2 shows no changes associated to histological grade. It is concluded that increased SNAIL levels in advanced PC are associated with low expression of syndecan 1. The mechanism by which SNAIL regulates the expression of syndecan 1 remains to be investigated.

## Introduction

Prostate cancer (PC) is the second most frequently diagnosed cancer in men worldwide. According to epidemiological data, the estimated new cases will be over 900,000 and estimated deaths over 250,000 each year (1).

PC originates from glandular epithelial cells mainly from the peripheral zone of the gland (2–4). During PC progression, normal tissue architecture is lost and malignant cells acquire invasive characteristics (5,6). In addition, PC is multifocal exhibiting different histopathological patterns graded from 1 to 5 (Gleason grades). Diagnosis is accompanied by Gleason score that considers the two predominant patterns, giving a final value ranging from 2–10, where high Gleason scores correspond to more undifferentiated tumors (7). This transformation involves alterations in cell morphology and function, called epithelial-mesenchymal transition (EMT) (8). During EMT many molecules change their expression pattern. Transcription factors such as SNAIL, SLUG and TWIST, and the mesenchymal markers N-cadherin and vimentin, increase their expression. Some adhesion molecules such as E-cadherin decrease their expression and others such as β-catenin change their location from the plasma membrane to the nucleus (9). It has been shown that the decrease in E-cadherin is associated with poor prognosis in various human tumors (10–13). In addition, E-cadherin overexpression in cultured cells and *in vivo* tumor models leads to a decrease of invasiveness and metastasis (14). Immunohistochemical studies on PC tissue microarray showed that SNAIL staining is associated with Gleason grade (15) with increasing expression from benign prostatic hyperplasia (BPH) to PC bone metastasis (16). SNAIL transcription factor is a zinc finger protein that can mediate EMT through downregulation of cell adhesion molecules such as E-cadherin by binding to E-boxes located in the gene promoter region. SNAIL can also lead to repression of tight junction proteins like claudin, occluidin and zona occluden-1 (ZO-1) (16).

Recently, syndecans, a heparan sulfate proteoglycan family, have been shown to be involved in the PC progression (17). In particular, syndecans 1 and 2 expression has been associated with the malignancy grade rated by the Gleason score (18–21). Transcriptional regulation of syndecans is poorly understood. A complete characterization of syndecan 1 and 2 promoters has been reported (22). In this regard, Vihinen *et al* (1996) were able to map a highly active syndecan 1 promoter region with binding capacity for Sp1 (22). No enhancer sites were found in either the upstream region or the first intron (up to +15 kb), while some repressor elements upstream of the promoter (−2.4 to −4 to 4 kb) were identified. In addition, 5 E-box sequences were found in syndecan promoter to which SNAIL might bind, repressing this syndecan in a direct way (23). Previous *in silico* analysis performed in our laboratory (unpublished data) revealed the presence of several putative binding sites for SNAIL-1 in the promoter regions of syndecans 1 and 2. The aim of this study was to evaluate the presence of SNAIL and its association with syndecans 1 and 2, and other EMT markers in PC samples and cell lines. We propose that syndecans may be regulated by SNAIL decreasing their expression during EMT in PC.

## Materials and methods

### Biopsy samples

PC specimens were obtained from the biopsy archive of the Pathological Anatomy Service, Clinic Hospital University of Chile, with the corresponding authorization of the institutional Ethics Committee. All samples were evaluated by an expert pathologist (I.G.). For the immunohistochemical evaluation specimens were grouped as BPH samples, a non-malignant control, and PC samples with high and low histological Gleason grade.

### Cell lines and culture conditions

Human PC cell lines (PC3 and LNCaP) were obtained from the American Type Culture Collection (ATCC, Rockville, MD, USA). Cells were cultured in Dulbecco’s modified Eagle’s medium (DMEM) supplemented with 10% fetal bovine serum and 1% penicillin and streptomycin. Cells were maintained under standard culture conditions at 37°C and 5% CO_2_ in a humidified environment.

### Antibodies

Primary antibodies were obtained from Abcam (SNAIL, N-cadherin; Cambridge, MA, USA), BD Transduction (E-cadherin; Franklin Lakes, NJ, USA), Santa Cruz Biotechnology (syndecan-1, Santa Cruz, CA, USA) and Contreras *et al* (24) (syndecan-2). Anti-rabbit secondary fluorescein-conjugated antibody, anti-mouse and anti-rabbit secondary peroxidase-conjugated antibodies were purchased from Jackson ImmunoResearch (West Grove, PA, USA).

### Tissue microarray (TMA) construction

The PC TMA was constructed as follows: first, the most representative tumor areas were carefully selected and marked based on the matched hematoxylin and eosin (H&E) stained slides. Altogether, 104 cores (1.5 mm diameter) of test tissue were taken from the donor blocks with a tissue microarrayer (Beecher Instruments, Silver Spring, MD, USA). Sections were stained with H&E and then evaluated by the pathologist. The TMA contained a mixture of tissue so that both benign and malignant samples of different Gleason grades were represented on each block. Sections of 4 *μ*m were obtained with a microtome and transferred to glass slides (SuperFrosts Plus, Menzel-Gläser, Braunschweig, Germany). Finally, colon (3) and tonsil (3) samples were included as positive control for syndecans.

### Immunohistochemistry

Tumor and control formalin-fixed and paraffin-embedded samples (TMA) were cut into 4-*μ*m sections, mounted, deparaffinized and rehydrated in decreasing concentration of ethanol and distilled water. The sections were washed with phosphate-buffered saline (PBS) and antigen retrieval was performed in a steam bath for 15 min at 90–95°C in 10 mM Citrate Buffer (pH 6.0). Endogenous peroxydase activity was inhibited by incubation in 3% H_2_O_2_. Later on, the sections were washed and non-specific binding was blocked with 10% normal horse serum solution (Vectastain). Then, sections were incubated with corresponding primary antibodies overnight at 4°C or 1 h at 37°C. Afterwards, samples were incubated with secondary antibody for 30 min at 37°C. Then, samples were washed and incubated with the streptavidin-biotin system (Histostain^®^-Plus Bulk Kit). After washing, the sections were incubated for 2 min at room temperature with liquid 3,3’-diaminobenzidine substrate (DAB) (Zymed^®^, LAB-SA Detection System and DAB-Plus Substrate Kit) followed by counterstaining with hematoxylin. Finally, samples were dehydrated in ethanol, cleared in xylene, coverslipped and evaluated in a microscope Leica DM 2500 (18,21).

### Immunocytochemistry

Cells were grown on 6-well tissue culture plates over sterilized glass coverslips until 50–70% confluence was reached. Then, cells were fixed with a solution containing 4% (v/v) paraformaldehyde and sucrose in PBS for 30 min at room temperature and stored in 0.02% (w/v) sodium azide in PBS at 4°C. Before incubation with the antibodies, the coverslips were washed with a 20 mM PBS-glycine solution and then blocked with PBS-glycine (20 mM)-BSA (0.1%). The cells were incubated with the primary antibodies overnight at 4°C or 1 h at 37°C, rinsed with 20 mM PBS-glycine solution three times and incubated with a FITC-conjugated secondary antibody (Jackson ImmunoResearch Fluorescein-Conjugated AffiniPure Goat Anti-Rabbit) away from light for 2 h. Finally, the coverslips were mounted and visualized under a spinning disc microscope (Olympus BX61Wl).

### Staining quantification

Photographs from immunohistochemistry and immunocytochemistry were digitally processed to obtain the integrated optical density (IOD). The average gray value of each image was used to obtain the IOD. The IOD corresponds to absorption of an optical element per unit distance for a given wavelength. The staining and illumination conditions of the samples were equivalent.

### Western blot analysis

The cell culture medium was aspirated and the cells were washed with PBS, trypsinized and centrifuged at 1,050 × g for 5 min. The resulting pellet was resuspended in a lysis buffer (50 nM Tris-HCl pH 7.4, 0.15 M NaCl, 1% sodium deoxycholate, 1% NP-40, 0.1% SDS, 5 nM EDTA), with a protease inhibitor cocktail (0.01 mg/ml benzamidine, 0.002 mg/ml antipain, 0.005 mg/ml leupeptin, 4 mM phenylmethylsulfonyl fluoride and 1 mM Na_3_VO_4_, pH 7.4). Later, the cells were scraped and the lysate was collected in a microfuge tube and passed through a syringe to break up the cell aggregates. The cell lysate was cleared by centrifugation at 15,000 × g for 15 min at 4°C, and the supernatant was discarded. The protein pellet was collected for protein quantification by the Bradford method at 570 nM using a Ray Leigh spectrophotometer (UV-1600 model). For western blot analysis, 40 *μ*g of protein were resolved over 10% polyacrylamide gels and electrotransferred onto a nitrocellulose membrane (Bio-Rad, Hercules, CA, USA). A molecular weight standard (Pierce, Rockford, IL, USA) was also resolved for analyzing specific zones of the gels. The efficiency of the process was measured staining the membranes with Ponceau Red reactive. The non-specific sites on membranes were blocked with blocking buffer [TBS-Tween-20 (100 mM Tris-HCl, 0.9% NaCl, 0.1% Tween-20, pH 7.5) - 5% non-fat dry milk] for 1 h at room temperature. Then, membranes were incubated with the corresponding primary antibody in blocking buffer overnight at 4°C, followed by incubation with anti-mouse or anti-rabbit secondary antibody peroxidase conjugated (in blocking buffer) and detected by chemiluminescence (Biological Industries, Beit Haemek, Israel) and autoradiography. The western blot analysis bands were scanned and analyzed using the scientific software program UN-SCAN-IT (Silk Scientific Corporation, Orem, UT, USA).

### Statistics analysis

Data were tabulated and analyzed using SPSS v17.0 software. Normal distribution was tested by Kolmogorov-Smirnov test. Given the distribution of the data, a parametric test (Pearson test) to calculate the correlation index was used. ANOVA (Tukey’s test) was used to compare means. P<0.01 was considered to indicate a statistically significant difference.

## Results

### TMA analysis

**Figure 1.**
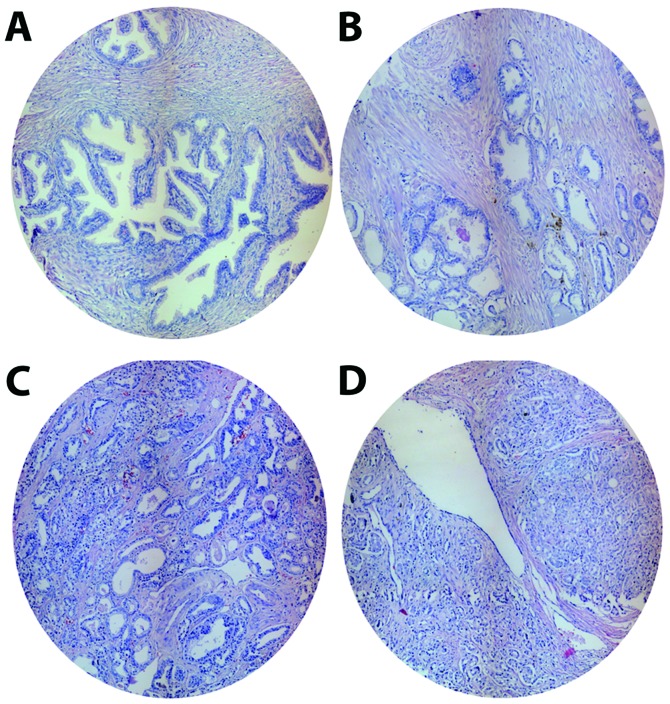
Representative spots included in micro tissue array (MTA) from prostate cancer samples. After histopathologic evaluation 4 groups of samples were distinguished within the MTA. (A) Benign prostatic hyperplasia (BPH); (B) low Gleason grade (LGG); (C) medium Gleason grade (MGG); and (D) high Gleason grade (HGG). Spot diameter, 1.5 mm.

From the 98 samples of PC in the TMA (excluding colon and tonsil controls), 4 spots containing prostatic stromal tissue were ruled out. Samples used for analysis were classified into 4 groups: non-tumoral control (BPH), and low, medium and high Gleason grade PC samples. The histological characteristics of the TMA groups stained with H&E are presented in Fig. 1. TMA included 45 BPH and 47 PC spots [9 corresponding to low (grade 1–2), 23 medium (grade 3) and 15 high Gleason grade (grade 4–5)], giving a total of 98 samples.

### SNAIL expression and distribution in prostate samples

**Figure 2.**
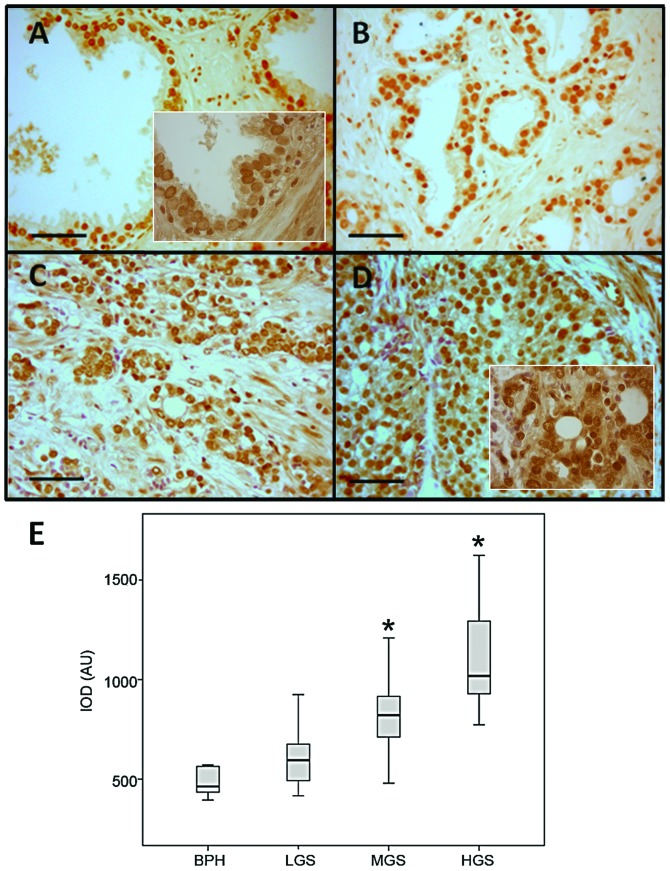
SNAIL expression and distribution in prostate samples. Immunostaining of SNAIL in samples of different histological grades. (A) Benign prostatic hyperplasia (BPH); (B) low Gleason grade (LGG); (C) medium Gleason grade (MGG); and (D) high Gleason grade (HGG). Inserts, ×1,000. (E) Quantification of SNAIL immunostaining. IOD, integrated optical density. AU, arbitrary units. ^*^P<0.01. Scale bar, 50 *μ*m.

SNAIL staining is observed mainly in nuclei and shows increased intensity in high Gleason compared to low grade samples (Fig. 2A). H&E dyeing was omitted to avoid interfering with SNAIL nuclear specific staining. Average IOD for each sample showed normal distribution (Kolmogorov-Smirnov P=0.689). Subsequently, the IOD means were compared by ANOVA. Samples with a high Gleason grades show SNAIL-staining IOD means significantly higher (P<0.01) than samples with low Gleason grade and BPH (P<0.0001) (Fig. 2B). Given the normal distribution, the Pearson test established a correlation coefficient of 0.734 between the IOD and the Gleason grade.

### Syndecan 1 expression and distribution in prostate samples

**Figure 3.**
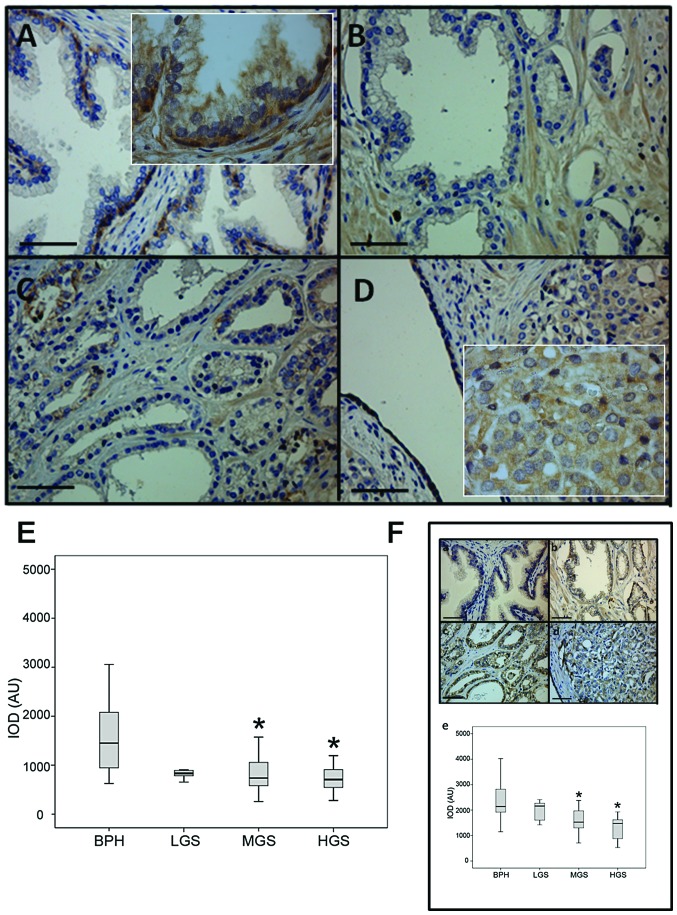
Syndecan 1 expression and distribution in prostate samples. Immunostaining of syndecan 1 in samples of different histological grades. (A) Benign prostatic hyperplasia (BPH); (B) low Gleason grade (LGG); (C) medium Gleason grade (MGG) and (D) high Gleason grade (HGG). (E) Quantification of syndecan 1 immunostaining. (F) E-cadherin (epithelial control marker) immunostaining: (a) Benign prostatic hyperplasia (BPH); (b) low Gleason grade (LGG); (c) medium Gleason grade (MGG) and (d) high Gleason grade (HGG). (e) Quantification of E-cadherin immunostaining. Inserts ×1,000. IOD, integrated optical density. AU, arbitrary units. ^*^P<0.01. Scale bar, 50 *μ*m.

The expression and distribution of syndecan 1 show a very heterogeneous pattern within the groups studied. BPH spots show a strong intensity localized mainly in the cytoplasm and membrane of the basal cells. Furthermore, epithelial cells exhibit a preferential localization in the baso-lateral region and approximately 50% of the cytoplasmic localization is detected at variable intensity (weak to moderate). This syndecan 1 distribution is also found in the low Gleason group and, to a lesser extent, in the medium Gleason group. However, in PC spots with high Gleason grade, membrane localization is lost and a granular cytoplasmic localization with low intensity is observed (Fig. 3). The main difference of this marker among the groups is found in its location. For comparison, E-cadherin (a validated epithelial marker) expression and distribution was evaluated in PC TMA samples. This epithelial marker shows an expected membrane location in most samples with intensities varying from moderate to strong. In BPH spots, E-cadherin shows mainly baso-lateral location in gland epithelial cells and was absent in apical membrane. On the other hand, low Gleason grade samples show syndecan 1 intensity and distribution similar to BPH. However, in high Gleason grade spots, a loss of intensity associated to gland architecture disorganization is observed (Fig. 3E). In addition, E-cadherin distribution shows a mixed pattern including cytoplasm location (Fig. 3F). Significant decrease in E-cadherin expression is observed only in medium and high Gleason grade samples (Fig. 3F).

### Syndecan 2 expression and distribution in prostate samples

**Figure 4.**
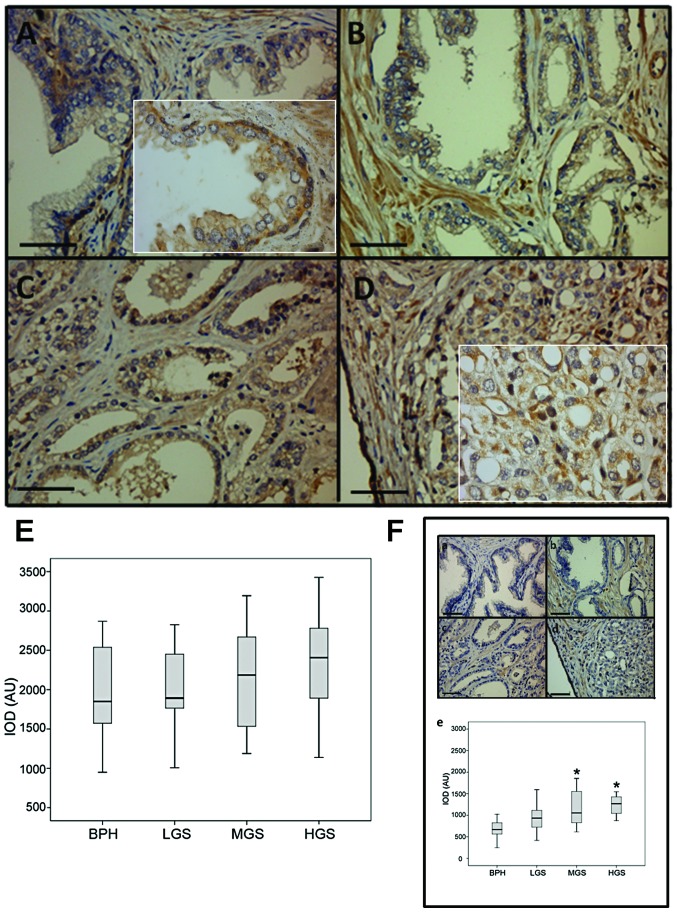
Syndecan 2 expression and distribution in prostate samples. Immunostaining of syndecan 2 in samples of different histological grades. (A) Benign prostatic hyperplasia (BPH); (B) low Gleason grade (LGG); (C) medium Gleason grade (MGG) and (D) high Gleason grade (HGG). (E) Quantification of syndecan 2 immunostaining. (F) N-cadherin (stromal control marker) immunostaining: a) Benign prostatic hyperplasia (BPH); b) low Gleason grade (LGG); c) medium Gleason grade (MGG) and d) high Gleason grade (HGG). e) Quantification of N-cadherin immunostaining. Inserts ×1,000. IOD, integrated optical density. AU, arbitrary units. ^*^P<0.01. Scale bar, 50 *μ*m.

The expression of this marker is highly variable in terms of location and immunostaining intensity. Similar to syndecan 1, syndecan 2 is found in both baso-lateral membrane and granular cytoplasm. BPH specimens show a high intensity in basal cells and basal lamina (Fig. 4A–D). No significant difference in syndecan 2 expression is observed among the different Gleason grade spots (Fig. 4E). However, cell location changes as Gleason grade increases, switching from membrane-cytoplasmic to cytoplasm-nucleus localization. Syndecan 2 is highly expressed in fibroblast, therefore, its presence in stroma served as internal positive control. For comparison, N-cadherin (a validated stromal marker) expression and distribution was evaluated in PC TMA samples. N-cadherin expression in BPH showed a mixed pattern including both membrane and cytoplasm location in epithelial cells. However, the immunostaining intensity, unlike E-cadherin, is weak (Fig. 4Fa–d). The intensity of N-cadherin staining is strong in stroma due to this molecule being highly expressed in fibroblast and mesenchymal tissue. For this reason, it serves also as an internal positive control (Fig. 4A). As expected, this marker is increasing with the disorganization of prostate gland epithelium. In low Gleason samples, N-cadherin is expressed mainly in baso-lateral membrane of epithelial cells while in medium grade spots the immunostaining of this molecule shows a decrease in membrane and an increase in cytoplasm. Furthermore, in high Gleason samples, N-cadherin shows a high expression of the membrane, cytoplasmatic and even nuclear location. N-cadherin expression is different only between BPH and medium/high Gleason grade samples (Fig. 4Fe).

### SNAIL expression in LNCaP and PC3 cell lines

**Figure 5.**
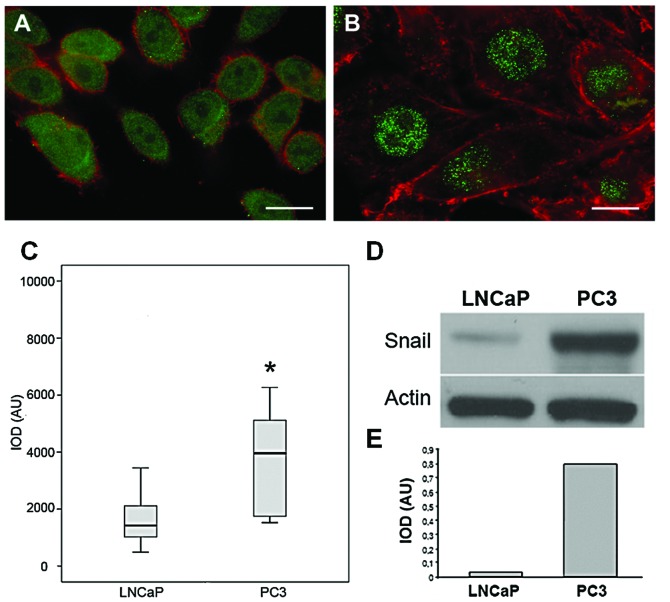
SNAIL expression and localization in LNCaP and PC3 cell lines. Flat-Z microphotographs in (A) LNCaP and (B) PC3 cell lines. SNAIL (green) and actin cytoskeleton (red). (C) Semi-quantification of SNAIL immunofluorescence in LNCaP and PC3 cell lines. (D) Western blot analysis of SNAIL in LNCaP and PC3 cell lines. (E) Densitometric analysis. IOD, integrated optical density. AU, arbitrary units. ^*^P<0.01. Scale bar, 50 *μ*m.

Considering that LNCaP and PC3 cell lines have been widely used as *in vitro* model for PC, we studied the location of the transcription factor SNAIL in these commercial cell lines using fluorescent immunocytochemistry. Different cellular SNAIL distribution is observed in these cell lines. LNCaP cells (low tumorigenic capacity) show a homogeneous localization in the nucleus and cytoplasm (Fig. 5A). However, PC3 cells (high tumorigenic capacity) show an exclusively nuclear localization (Fig. 5B). Furthermore, the SNAIL staining intensity is very high and occasionally detected at the perinuclear region. Localization is more evident when performing a merge between SNAIL staining (green) and actin microfilaments (red). When comparing IOD, significant differences in the SNAIL expression between the cell lines were found (Fig. 5C). In addition, protein extraction and western blot analysis were performed to compare the SNAIL expression between the cell lines. Results show a higher SNAIL protein expression in PC3 than LNCaP cells (Fig. 5D and E).

### Syndecans 1 and 2 expression in LNCaP and PC3 cell lines

**Figure 6.**
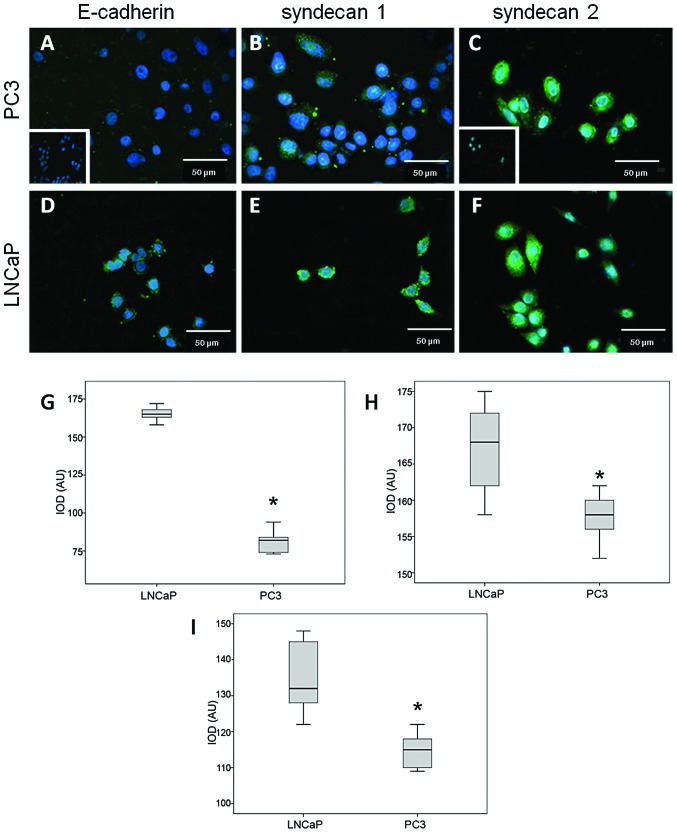
Syndecans 1 and 2 and E-cadherin ectodomain localization in LNCaP and PC3 cell lines. (A) E-cadherin ectodomain, (B) syndecan 1 and (C) syndecan 2 in PC3 cell line. (D) E-cadherin ectodomain, (E) syndecan 1 and (F) syndecan 2 in LNCaP cell line. Inserts, negative controls. DAPI for nuclear staining. Magnification, ×400. (G, H and I) Semi-quantification of syndecan 1, syndecan 2 and E-cadherin, immunofluorescence in LNCaP and PC3 cell lines, respectively. IOD, integrated optical density. AU, arbitrary units. ^*^P<0.01.

Results obtained from syndecans 1 and 2 expression are presented in Fig. 6. In LNCaP and PC3 cells, expression of both syndecans is evident at plasma membrane (Fig. 6B, C, E and F). Comparison of IOD, in LNCaP cells (low tumorigenic capacity) show a higher syndecans expression than PC3 cells (high tumorigenic capacity) (Fig. 6G and H). E-cadherin (control epithelial marker) shows a similar pattern (Fig. 6A and D) and IOD (Fig. 6I).

## Discussion

Searching for markers with diagnostic and prognostic utility is a major challenge in cancer field. In this regard, several markers of EMT such as SNAIL and TWIST, have recently been associated with clinical variables in localized PC. In this analysis, TWIST and vimentin, stand out as good predictors of biochemical recurrence (25). Recently, some roles for proteoglycans in PC have been reported. Cellular changes and enzymatic activity in the developing tumor can alter the composition and structure of proteoglycans modifying their function (17). Our group has reported that some heparan sulfate proteoglycans (syndecans 1 and 2) have a close association with malignancy and may also be useful as markers of biochemical recurrence of PC (18,21). Regarding syndecan 1, other studies have pointed out its utility as a marker of malignancy with prognostic utility. In these studies syndecan 1 is expressed in inverse relation to Gleason score (26,27). The prognostic value of this syndecan in patients treated with radical prostatectomy has been also established (28). However, other authors reported, despite the reduction of syndecan 1 in high Gleason samples, that this syndecan is not a good predictor for tumor recurrence or survival, reducing its clinical importance as a marker (29). Regarding syndecan 2, changes from membrane to cytoplasm localization are associated with increasing Gleason score. The syndecan 2 distribution is observed mainly at the cytoplasm and nucleus in high Gleason grades. Nuclear presence of this syndecan suggests its involvement in transcriptional processes. Our results are consistent with recent reports detecting nuclear localization of syndecans (30). In addition, the proteolytic cleavage of syndecan results in extracellular releasing of its ectodomain. Multiple roles have been described for syndecan shedding in health and disease (31,32). The ectodomain may promote tumor growth and angiogenesis (33) and cytosolic domain might be translocated to the nucleus regulating gene expression (30).

Recently, Smith and Odero-Marah (16) have reported the possible role of SNAIL in PC and its potential utility as a therapeutic target. Furthermore, it has been reported that the SNAIL1 increased expression was positively correlated with PC de-differentiation, but not with cancer progression or prognosis. There is evidence indicating that SNAIL expression is upregulated from the early stages of PC (15). The association between increased expression of SNAIL and prostate malignancy found in the present study is in agreement with other previous works (8,34). Evidence provided by this work support the hypothesis that SNAIL could be repressing the expression of syndecan 1, in the same way as E-cadherin (35,36). The decreased expression of syndecan 1 is associated with the loss of basal cells and normal epithelial organization. Considering that there are putative binding sites for SNAIL in both syndecans promoters, it is reasonable to suggest an active role for SNAIL in PC malignancy regulation. In our study, SNAIL was detected preferentially localized in the nuclear region showing a gradually increasing intensity with the Gleason grade. In addition, the high SNAIL expression in PC3 cells (high tumorigenic capacity) compared with LNCaP cells (low tumorigenic capacity), strongly suggest that SNAIL could be favoring the tumorigenic process through different cellular mechanisms. In PC cell lines, the expression of SNAIL, using specific siRNA, has been shown to play a role by inhibiting cellular aging (37). As a result, such cells decreased their survival, presenting an increase in caspase activity. Baritaki *et al* (38) studied the effects of a proteasome inhibitor (NPI-0052) on metastatic PC cell lines showing that treated cells decreased SNAIL levels and increased expression of E-cadherin. In addition, these cells were unable to initiate EMT, exhibiting a low degree of invasiveness.

According to our results, the positive correlation between high SNAIL expression and PC malignancy might be associated with metalloproteinases induction (expression or activation). These enzymes could be responsible for the proteolytic shedding of syndecans explaining the decrease in their immunohistochemical staining. Furthermore, the decreased expression of E-cadherin (repressed by SNAIL) and the elevated expression of N-cadherin would complete the model of PC progression.

On the contrary, it has been recently described in PC, that TNFα can stabilize SNAIL level favoring EMT (39). Thus, EMT may involve the coordinated upregulation of SNAIL and the downregulation of syndecans during PC progression.
